# Overexpressed Thrombospondin 2 Induced Osteogenic Differentiation of Valve Interstitial Cells via Inhibition of Akt/NF-*κ*B Signaling Pathway to Promote Calcific Aortic Valve Disease Development

**DOI:** 10.1155/2022/2022958

**Published:** 2022-09-08

**Authors:** Cheng Yu, Danna Wu, Chong Zhao, Chaoguang Wu

**Affiliations:** ^1^Department of Cardiac Surgery, Hainan General Hospital (Hainan Affiliated Hospital of Hainan Medical University), Haikou, Hainan Province 570311, China; ^2^Department of Pharmacy, Hainan General Hospital (Hainan Affiliated Hospital of Hainan Medical University), Haikou, Hainan Province 570311, China; ^3^College of English, Qiongtai Normal University, Haikou, Hainan Province 571127, China

## Abstract

Thrombospondin 2 (THBS2) is reported to participate in the development of calcific aortic valve disease (CAVD), while the effects are not elucidated completely. The study aimed to explore the role and mechanism of THBS2 in CAVD. Differentially expressed genes related to stenosis and sclerosis were screened through Limma package based on data from Gene Expression Omnibus (GEO), and the functional enrichment analysis was performed by the Database for Annotation, Visualization and Integrated Discovery (DAVID) database. The immunoreactivity of THBS2 in CAVD and normal samples was detected through immunohistochemistry. Valve interstitial cells (VICs) were transfected with short hairpin RNA against THBS2 (shTHBS2) and THBS2 overexpression plasmid and treated with LY294002 (Akt inhibitor) and induced osteogenic differentiation. The expression of THBS2 in CAVD and normal samples and the levels of THBS2, osteocalcin, Runx2, SPARC, COL1A2, COL1A1, SPP1, CTGF, MMP-2, MMP-13, Akt, p-Akt, p65, p-p65, and nuclear p65 in VICs were tested by qRT-PCR and Western blot. ALP activity was assessed using colorimetry. Calcic nodule formation was measured by Alizarin Red staining. THBS2 and PI3K-Akt pathway were differentially enriched in stenosis samples when compared with those in sclerosis samples. THBS2 expression was upregulated in CAVD and positively correlated with ALP activity, calcic nodule formation, osteogenic differentiation-related (osteocalcin, Runx2, SPARC, COL1A2, COL1A1, SPP1, and CTGF) and extracellular matrix– (ECM–) related (MMP-2 and MMP-13) factors in the process of osteogenic differentiation. ShTHBS2 suppressed ALP activity, calcic nodule formation, and osteogenic differentiation/ECM-related molecules while upregulating p-Akt/Akt, p-p65/p65, and nuclear p65 expressions in VICs during osteogenic differentiation. However, THBS2 overexpression had the opposite effect to shTHBS2, and LY294002 reversed the effect of shTHBS2. Collectively, overexpressed THBS2 induces the osteogenic differentiation of VICs via inhibiting Akt/NF-*κ*B pathway to promote the development of CAVD.

## 1. Introduction

Identified as a degenerative and progressive disease, calcific aortic valve disease (CAVD) is the most frequent heart valve disease contributing to a large part of cardiovascular disease incidence and death around the world [1–3]. Age, sex, tobacco smoking, type II diabetes mellitus, hypertension, and hypercholesterolemia are all risk factors associated with the increase of CAVD morbidity [2]. Owing to the rise of aging population in recent years, the incidence of CAVD is expected to elevate year by year [4]. CAVD is characterized by a variety of cellular events that cause aortic valve sclerosis (AVSc, known as the initial phase of CAVD), including fibrotic thickening and extensive aortic valve calcification, culminating in clinically serious aortic stenosis (AS), with surgical replacement as the only feasible therapeutic strategy at present [4–8]. Although treatment agents such as renin-angiotensin-aldosterone system regulators and HMG-CoA reductase inhibitors indeed improve multiple pivotal factors associated with CAVD *in vitro*, their efficacy has not been validated in clinical trials yet [4, 9]. Furthermore, the generally asymptomatic AVSc makes early diagnosis extremely hard, and certain efforts dedicated to preventing or reversing aortic cusp calcification and alleviating AS also turn out to be less than satisfactory [4, 10–12]. More and more researchers are working to find suitable diagnostic and therapeutic targets for CAVD [13], for example, it has been reported that miR-125b and CCL4 appear to be involved in the progression of CAVD and may offer novel therapeutic and diagnostic strategies related to this disease [14]; besides, calcium signaling pathway genes RUNX2 and CACNA1C are associated with CAVD [15]. Hence, there is an urgent need for exploring novel and effective diagnostic and therapeutic methods.

Thrombospondin 2 (THBS2), as a member of thrombospondin family composed of conserved proteins with regions of high sequence identity, is a matricellular glycoprotein which modulates diverse biological functions, including cell adhesion, extracellular matrix (ECM) remodeling, proliferation, angiogenesis, and inflammation [7, 16–19]. THBS2 has four protein-binding domains, comprising a von Willebrand factor-like domain, a globular C-terminal domain, an N-terminal heparin binding domain, and type I–III Thrombospondin repeats, which exerts its effects via interaction between those domains and different receptors of cell surface [20]. Previous studies have revealed controversial roles of THBS2 in distinct malignancies. For instance, lower-expressed THBS2 is related to the poor prognosis of patients with gastric cancer [21], cervical cancer [22], and ovarian cancer [23], while overexpressed THBS2 is related to the poor prognosis of patients with prostate cancer [24], lung cancer [25], and oral cancer [26]. What's more, an additional report has documented that the high-expressed THBS2 possibly participate in inflammation, ECM remodeling, and neovascularization during CAVD advancement [7]. However, the effects and detailed mechanism concerning the effects of THBS2 on CAVD have not been fully expounded up to now.

Valve interstitial cells (VICs) are the major cell type composing aortic valve (AV), and their phenotypic transformation into osteoblast-like VICs is reported to play a positive role in the progression of CAVD by osteogenic trans-differentiation and calcium deposition (calcification) [3, 5, 8, 27, 28]. As such, approaches that efficiently repress the osteogenic differentiation of VICs to prevent their transition may provide some new insights for the treatment of CAVD [29]. The present work, accordingly, investigated the impact of THBS2 upon the calcification of VICs and its specific mechanism.

## 2. Materials and Methods

### 2.1. Bioinformatics Analysis

The gene expression profile Series GSE138531, including data of valve samples from patients with asymptomatic AVSc and severe AS, was downloaded from Gene Expression Omnibus (GEO, http://www.ncbi.nlm.nih.gov/geo/). Limma package was employed to screen differentially expressed genes (DEGs) in AVSc and AS, for which the functional enrichment analysis was performed through Database for Annotation, Visualization and Integrated Discovery (DAVID) [30].

### 2.2. Human samples

The AV tissues and adjacent normal tissues were acquired from 25 patients with CAVD who received the aortic valve replacement (AVR) in Hainan General Hospital from April 2018 to August 2020. Primary human normal noncalcified AV cells were obtained during the cardiac transplant. All enrolled patients had signed written informed consent prior to the research. The clinical trial program was reviewed and approved by the Ethics Committee of Hainan General Hospital (HN20180422307).

### 2.3. Immunohistochemistry

Following the fixation in 10% neutral formalin fix solution (E672001, Sangon Biotech Co., Ltd., Shanghai, China) at 4 °C overnight, the tissue samples were dehydrated and embedded in paraffin. Subsequently, serial 5-*μ*m-thick tissue sections were obtained by a Leica RM2235 automated microtome (Leica Microsystems, Wetzlar, Hesse-Darmstadt, Germany, https://www.leica-microsystems.com.cn/cn/). After deparaffinage in xylene (C_8_H_10_, ≥75.0%; 214736, Sigma-Aldrich, St. Louis, MO, USA) and hydration in gradient ethanol (C_2_H_6_O; 1.00983, Sigma-Aldrich, USA), the sections were washed with phosphate buffer saline (PBS; P1022, Beijing Solarbio Science & Technology Co., Ltd., Beijing, China) and permeabilized using immunostaining permeabilization solution with Triton X-100 (P0096, Beyotime Biotechnology, Shanghai, China) at 37 °C for 10 minutes (min). Subsequently, the sections were rinsed by PBS, blocked in endogenous peroxidase blocking buffer (P0100A, Beyotime Biotechnology, China) at room temperature for 10 min, and washed again with PBS. Citrate Antigen Retrieval Solution (E673001, Sangon Biotech Co., Ltd., China) was applied to retrieve antigen, followed by an incubation with 5% goat serum (ab7481, Abcam, Cambridge, MA, USA) at 37 °C for 30 min in a moist chamber. After the removal of serum, the sections were cultured with the primary rabbit polyclonal antibody against THBS2 (1:1000; ab112543, Abcam, USA) at 4 °C overnight. The sections were then rewarmed at 37 °C for 45 min, rinsed by PBS, and incubated with the corresponding secondary antibody: goat anti-rabbit IgG H&L Horseradish Peroxidase (HRP) (1:2000; ab205718, Abcam, USA) at 37 °C for 30 min. A DAB Horseradish Peroxidase Color Development Kit (E670033, Sangon Biotech Co., Ltd., China) was used for color reaction according to the instructions. Next, the sections were immersed in hematoxylin staining solution (E607317, Sangon Biotech Co., Ltd., China) for 20 seconds (s), washed with water, and immersed in PBS for 5 min to make the color of nucleus back to blue. Finally, after dehydration and transparency mounting, the sections were observed under a Leica DM2500 LED optical microscope (Leica Microsystems, Germany) at the magnification of ×200.

### 2.4. Cell Culture and Induction of Osteogenic Differentiation

Human noncalcified aortic valves with normal echocardiographic analyses were harvested during cardiac transplant surgery to construct human valve interstitial cells (VICs). VICs were incubated in Dulbecco's Modified Eagle Medium (DMEM; PM150210A, Procell Life Science & Technology Co., Ltd., Wuhan, China, https://www.procell.com.cn/) supplemented with 10% fetal bovine serum (FBS; 10100147C, Thermo Fisher Scientific, Waltham, MA, USA) at 37 °C in a humidified atmosphere with 5% CO_2_. Cells at subculture 5-10 were adopted in the present research.

In order to induce osteoblast differentiation, VICs were cultured in *α*-Minimum Essential Medium (*α*-MEM; 12571048, Thermo Fisher Scientific, USA) blended with 100 nmol/L dexamethasone (C_22_H_29_FO_5_, ≥97%; D4902, Sigma-Aldrich, USA), 50 ng/mL BMP-2 (≥98%; SRP3326, Sigma-Aldrich, USA), 50 mg/mL ascorbic acid (C_6_H_8_O_6_; 1043003, Sigma-Aldrich, USA), 0.1% FBS, and 5 mmol/L *β*-glycerophosphate (C_3_H_7_O_6_PNa_2_ · xH2O,≥99%; G9422, Sigma-Aldrich, USA) for 14 days (d). The VICs of control group were cultured in normal medium [3].

### 2.5. Cell transfection and Treatment

The overexpression vector (pcDNA3.1/+vector; V79020, Invitrogen, Thermo Fisher Scientific, USA) carrying THBS2 gene and short hairpin RNA against THBS2 (shTHBS2; 5′-CCGGCAAGTACGAATGCAGAGATATCTCGAGATATCTCTGCATTCGTACTTGTTTTTG-3′, Guangzhou RiboBio Co., Ltd., Guangzhou, China) were transfected into VICs to establish the overexpressed and silenced gene models using Lipofectamine 2000 Transfection Reagent (11668500, Thermo Fisher Scientific, USA), while the empty vector was used as the negative control (vector). Untreated cells was used as the control group.

For transfection, briefly, VICs were digested with trypsin (C0205, Beyotime Biotechnology, China), seeded at a density of 1 × 10^5^ cells/well in a 24-well plate, and incubated until 70-90% confluence was reached. Following the dilution of Lipofectamine 2000 (2.0 *μ*L) in non-serum Opti-MEM Medium (50 *μ*L; 31985062, Thermo Fisher Scientific, USA) at room temperature for 5 min and DNA (0.8 *μ*g) in Opti-MEM Medium (50 *μ*L), the two dilutions were mixed together and cultured at room temperature for 20 min, subsequent to which the complexes (100 *μ*L/well) were added into cells and the plate was shaken back and forth for a thorough mixture. Cells were cultured in a CO_2_ incubator at 37 °C for 24 or 48 hours (h). To assess the effect of THBS2 on the osteogenic differentiation of VICs, transfected cells were cultured under the induction of osteogenic differentiation for 7 d. For the evaluation of signaling pathway, cells transfected with shTHBS2 were treated with 10 *μ*M Akt inhibitor LY294002 (C_19_H_17_NO_3_; 19-142, Sigma-Aldrich, USA) for 24 h [31].

### 2.6. Quantitative Reverse Transcription-Polymerase Chain Reaction (qRT-PCR)

A D-500 homogenizer (Wiggens, Straubenhardt, Germany, http://www.wiggens.com/) was utilized for tissue grinding prior to the extraction of total RNA from tissues and cells with TRIeasyTM Total RNA Extraction Reagent (10606ES60, Yeasen Biotech Co., Ltd., Shanghai, China, http://www.yeasenbiotech.com/). The nuclear mRNA was isolated by Cytoplasmic & Nuclear RNA Purification Kit (NGB-37400, NorgenBiotek, AmyJet Scientific Inc., Wuhan, China, https://www.amyjet.com/). The cDNA was synthesized through reverse transcription using Hifair® III 1st Strand cDNA Synthesis SuperMix for qPCR (gDNA digester plus) (11141ES10, Yeasen Biotech Co., Ltd., China). The cDNA was amplified in ABI 7500 real-time fluorescence quantitative PCR instrument (Thermo Fisher Scientific, USA) under the indicated conditions, including the predenaturation at 95 °C for 30 s, followed by 40 cycles of 95 °C for 3 s and 60 °C for 32 s, which was traced by Hieff UNICON® Universal Blue qPCR SYBR Green Master Mix (11184ES03, Yeasen Biotech Co., Ltd., China). The sequences of primers (Guangzhou RiboBio Co., Ltd., China) are provided in Table 1. Glyceraldehyde-3-phosphate dehydrogenase (GAPDH) and Lamin B1 were the loading controls. The 2^-*ΔΔ*CT^ relative quantification method was adopted for the calculation of data [32].

### 2.7. Western Blot

Radio immunoprecipitation assay (RIPA) buffer (P0013E, Beyotime Biotechnology, China) was employed for extracting total protein. Following the centrifugation at 14000×*g* for 3 min at 4 °C, the nuclear protein was extracted through Nuclear and Cytoplasmic Protein Extraction Kit (P0028, Beyotime Biotechnology, China). The protein concentration was determined using BCA Protein Assay Kit (P0011, Beyotime Biotechnology, China). Equal amounts of protein (45 *μ*g) and marker (5 *μ*L; PR1910, Beijing Solarbio Science & Technology Co., Ltd., China) were separated by 6-8% SDS polyacrylamide gel electrophoresis (SDS-PAGE, P0012A, Beyotime Biotechnology, China), subsequent to which the protein sample was transferred onto the PVDF membranes (88585, Thermo Fisher Scientific, USA). After being blocked in 5% nonfat milk for 1 h, the PVDF membranes were incubated at 4 °C overnight with the primary antibodies against THBS2 (rabbit, 1:1000; ab112543, Abcam, USA), phosphorylation (p)-AKT (rabbit, 1:1000; ab38449, Abcam, USA), Akt (rabbit, 1:500; ab8805, Abcam, USA), p65 (rabbit, 1:2000; ab16502, Abcam, USA), p-p65 (rabbit, 1:2000; ab86299, Abcam, USA), GAPDH (rabbit, 1:10000; ab181602, Abcam, USA), and Lamin B1 (rabbit, 1:10000; ab16048, Abcam, USA). Next, the membranes were rinsed by Tris-buffered saline containing Tween 20 (TBST; ST673, Beyotime Biotechnology, China), cultured with corresponding HRP-conjugated secondary antibody anti-rabbit IgG H&L (ab6721, 1:5000; Abcam, USA) at room temperature for 1 h, and washed again with TBST. The BeyoECL Plus (P0018M, Beyotime Biotechnology, China) was applied to visualize protein in e-BLOT chemiluminescence Imager (GUANGZHOU ESHINE INSTRUMENT, Guangzhou, China, http://www.eshinesci.com/). Finally, the data were analyzed by Image J (version 1.48, National Institutes of Health, Bethesda, MD, USA).

### 2.8. Alkaline Phosphatase (ALP) Activity Assay

The ALP activity of cells was tested by Alkaline Phosphatase Assay Kit (P0321M, Beyotime Biotechnology, China). Specifically, cells were lysed using cell lysis buffer for Western and IP without inhibitors (P0013J, Beyotime Biotechnology, China). The cell lysate, chromogenic substrate, and detection buffer were well-mixed in a 96-well plate and incubated at 37 °C for 10 min. Following the addition of the stop solution, the absorbance at 405 nm was measured using a microplate reader (Varioska LUX, Thermo Fisher Scientific, USA).

### 2.9. Alizarin Red Staining

Harvested VICs were rinsed three times with PBS, fixed in 95% ethanol for 10 min, and washed again three times with PBS. Subsequently, 0.2% Alizarin Red S Staining Solution (C0140, Beyotime Biotechnology, China) was added into cells, followed by an incubation at 37 °C for 30 min. After being rinsed with distilled water, cells were viewed under a microscope at the magnification of ×100 and photographed.

### 2.10. Statistical Analysis

Statistical analysis was achieved by GraphPad Prism 8.0 (GraphPad Software Inc., San Diego, CA, USA). All data were collected from independent experiments repeated at least triplicate and expressed as mean ± standard deviation. The level of THBS2 in CAVD and normal adjacent tissues was compared by paired-samples *t*-test, while one-way ANOVA with Dunnett's or Tukey's post hoc test was used for comparison among multiple groups. *p* < 0.05 was considered to be statistically significant.

## 3. Results

### 3.1. THBS2 Expression Was Upregulated in CAVD and Related to Osteogenic Differentiation of VICs

**Figure 1:**
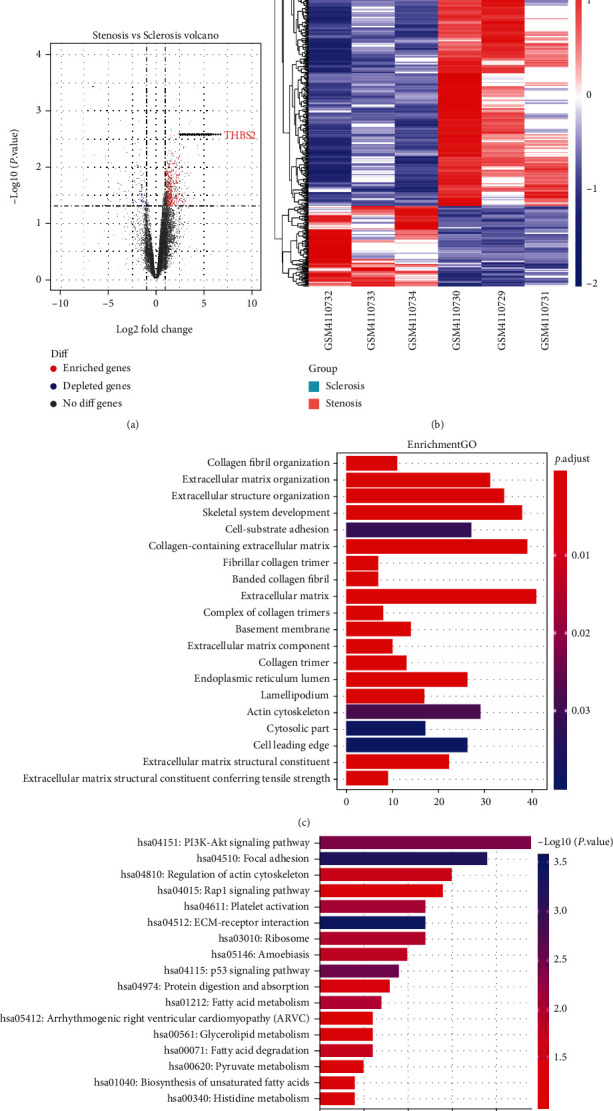
DEGs in stenosis and sclerosis samples were identified through bioinformatics. (a) Volcano plot of DEGs in stenosis and sclerosis samples. (b) Heatmap of DEGs in stenosis and sclerosis samples. (c) and (d) Bar graph of functional enrichment analysis performed through DAVID. Abbreviation: DEGs: differentially expressed genes.

**Figure 2:**
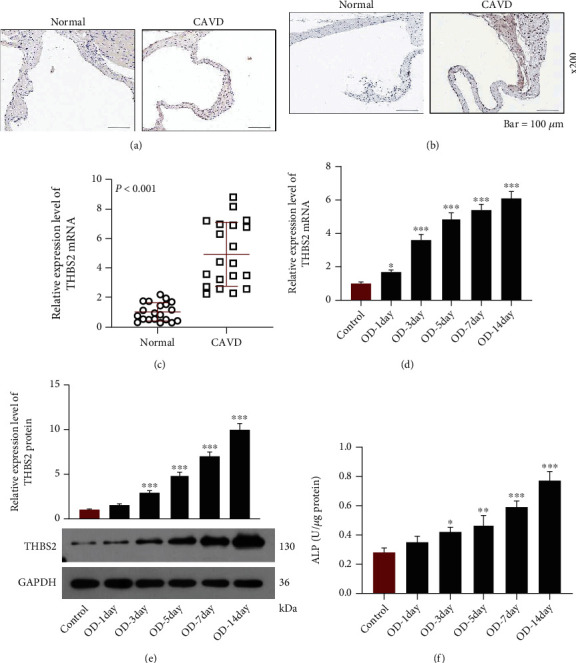
THBS2 was high-expressed in CAVD and related to increased ALP activity during the OD of VICs. (a) and (b) The immunoreactivity of THBS2 in AV endothelial cells (a) and calcified AV tissue (b) from normal and CAVD samples was evaluated by immunohistochemistry (scale: 100 *μ*m; magnification: ×200). (c) Relative mRNA expression of THBS2 in CAVD and adjacent normal tissues was assessed through qRT-PCR. GAPDH was a loading control. (d and e) Relative mRNA (d) and protein (e) expressions of THBS2 in VICs during the process of OD were tested by qRT-PCR and Western blot. GAPDH was a loading control. (f) ALP activity of VICs during the process of OD was detected through colorimetry. ^∗^*p* < 0.05, ^∗∗^*p* < 0.01, ^∗∗∗^*p* < 0.001 vs. control group. All experiments were repeated independently at least three times. Data were expressed as the means ± standard deviation. Abbreviations: THBS2: thrombospondin 2; CAVD: calcific aortic valve disease; ALP: alkaline phosphatase; OD: osteogenic differentiation; VICs: valve interstitial cells; AV: aortic valve; qRT-PCR: quantitative reverse transcription-polymerase chain reaction; GAPDH: glyceraldehyde-3-phosphate dehydrogenase.

As shown in Figure 1, there were total 736 DEGs in the samples of stenosis and sclerosis, among which THBS2 was markedly higher-expressed in stenosis samples as compared with sclerosis samples. PI3K-Akt signaling pathway was found to be involved in CAVD (Figure 1(d)). The result of immunohistochemistry indicated that compared with normal samples, the stronger immunoreactivity of THBS2 was evidenced in both AV endothelial cells and calcified AV tissue of CAVD samples (Figures 2(a) and 2(b)). In addition, the result of qRT-PCR exhibited that the expression of THBS2 in AV tissues from CAVD patients was appreciably higher than that of adjacent normal tissues (Figure 2(c), *p* < 0.001). These data underlined that THBS2 was highly expressed in CAVD, further validating the results acquired in aforementioned bioinformatics.

**Figure 3:**
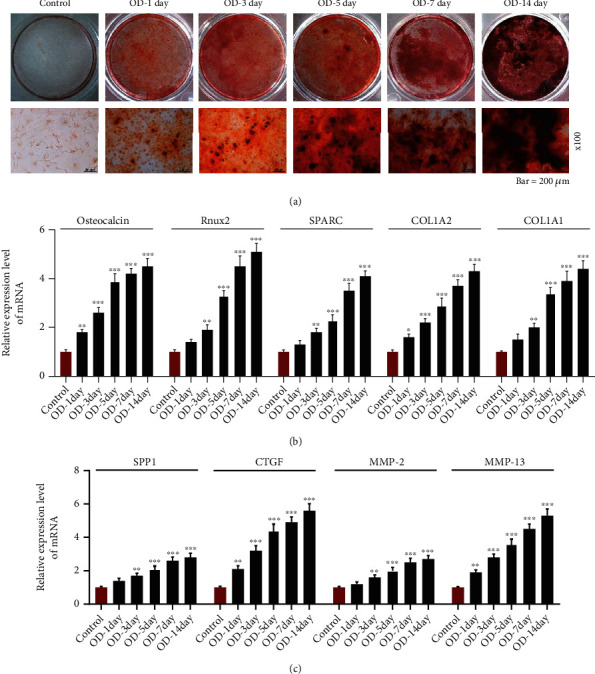
THBS2 was related to increased calcic nodule formation and the expressions of factors associated with OD and ECM during the OD of VICs. (a) Calcic nodule formation of VICs during the process of OD was observed by Alizarin Red staining (scale: 200 *μ*m; magnification: ×100). (b) and (c) Relative mRNA expressions of osteocalcin, Runx2, SPARC, COL1A2, and COL1A1 (b) and SPP1, CTGF, MMP-2, and MMP-13 (c) in VICs during the process of OD were determined through qRT-PCR. GAPDH was a loading control. ^∗^*p* < 0.05, ^∗∗^*p* < 0.01, ^∗∗∗^*p* < 0.001 vs. control group. All experiments were repeated independently at least three times. Data were expressed as the means ± standard deviation. Abbreviations: THBS2: thrombospondin 2; OD: osteogenic differentiation; ECM: extracellular matrix; VICs: valve interstitial cells; Runx2: Runt-related transcription factor-2; SPARC: secreted protein acidic and rich in cysteine; COL1A: collagen type I alpha; SPP1: secreted phosphoprotein 1; CTGF: connective tissue growth factor; MMP-: matrix metalloproteinase-; qRT-PCR: quantitative reverse transcription-polymerase chain reaction; GAPDH: glyceraldehyde-3-phosphate dehydrogenase.

With the progression of osteogenic differentiation, at the indicated time points, the obviously elevated mRNA and protein levels of THBS2 were observed in VICs (Figures 2(d) and 2(e), *p* < 0.05), together with the increase on ALP activity (Figure 2(f), *p* < 0.05), colored area of Alizarin staining (Figure 3(a)), and the mRNA expressions of osteocalcin, Runt-related transcription factor-2 (Runx2), secreted protein acidic and rich in cysteine (SPARC), *α*2 chain of type I collagen (COL1A2), COL1A1, secreted phosphoprotein 1 (SPP1), connective tissue growth factor (CTGF), matrix metalloproteinase (MMP)-2, and MMP-13 (Figures 3(b) and 3(c), *p* < 0.05). These results thus suggested that THBS2 might be associated with the osteogenic differentiation of VICs.

### 3.2. THBS2 Mediated the Osteogenic Differentiation of VICs

**Figure 4:**
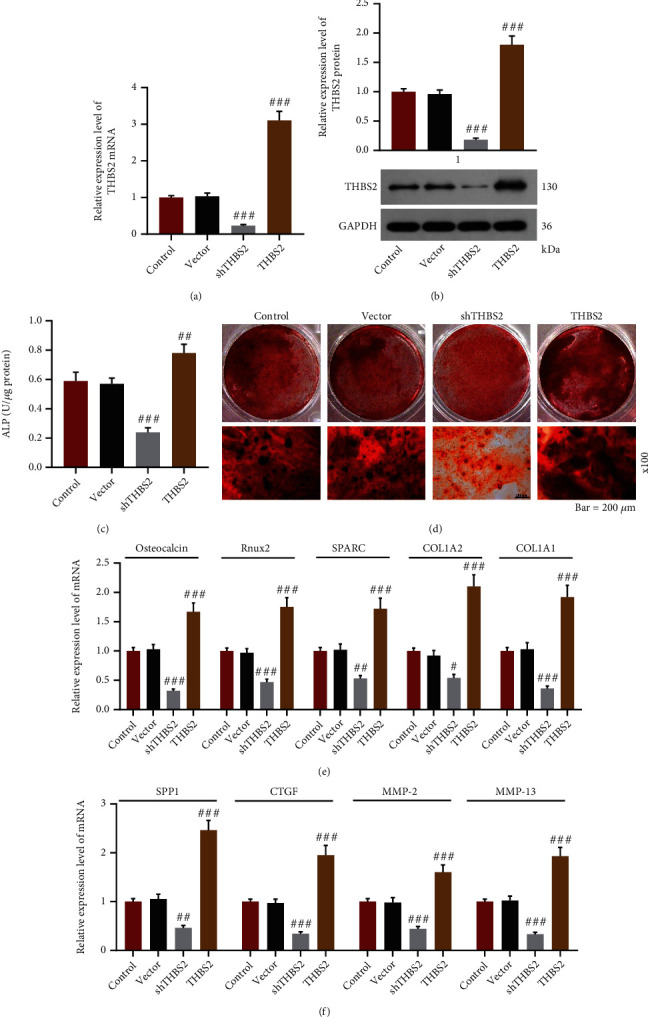
THBS2 mediated OD of VICs. (a) and (b) Relative mRNA (a) and protein (b) expressions of THBS2 in VICs transfected with shTHBS2 and THBS2 overexpression plasmid were evaluated by qRT-PCR and Western blot after the induction of OD. GAPDH was a loading control. (c) ALP activity of VICs transfected with shTHBS2 and THBS2 overexpression plasmid was detected through colorimetry after the induction of OD. (d) Calcic nodule formation of VICs transfected with shTHBS2 and THBS2 overexpression plasmid was observed by Alizarin red staining after the induction of OD (scale: 200 *μ*m; magnification: ×100). (e and f) Relative mRNA expressions of osteocalcin, Runx2, SPARC, COL1A2, and COL1A1 (e) and SPP1, CTGF, MMP-2, and MMP-13 (f) in VICs transfected with shTHBS2 and THBS2 overexpression plasmid were determined through qRT-PCR after the induction of OD. GAPDH was a loading control. ^#^*p* < 0.05, ^##^*p* < 0.01, ^###^*p* < 0.001 vs. vector group. All experiments were repeated independently at least three times. Data were performed as the means ± standard deviation. Abbreviations: THBS2: thrombospondin 2; OD: osteogenic differentiation; VICs: valve interstitial cells; shTHBS2: short hairpin THBS2; qRT-PCR: quantitative reverse transcription-polymerase chain reaction; GAPDH: glyceraldehyde-3-phosphate dehydrogenase; ALP: alkaline phosphatase; Runx2: Runt-related transcription factor-2; SPARC: secreted protein acidic and rich in cysteine; COL1A: collagen type I alpha; SPP1: secreted phosphoprotein 1; CTGF: connective tissue growth factor; MMP-: matrix metalloproteinase-.

To further investigate the effect of THBS2 on the osteogenic differentiation of VICs, VICs were transfected with shTHBS2 and THBS2 overexpression plasmid before the induction of osteogenic differentiation. In comparison with the empty vector group, a prominent downregulation of THBS2 was observed in shTHBS2 group (Figures 4(a) and 4(b), *p* < 0.001), whereas a marked rise of THBS2 level was noticed in THBS2 group (Figures 4(a) and 4(b), *p* < 0.001), indicating a successful construction of silenced and overexpressed models. Contrasted with the vector group, the ALP activity, colored area of Alizarin staining, and the mRNA levels of osteocalcin, Runx2, SPARC, COL1A2, COL1A1, SPP1, CTGF, MMP-2, and MMP-13 were evidently lower in shTHBS2 group (Figures 4(c)–4(f), *p* < 0.05), while those were dramatically higher in THBS2 group (Figures 4(c)–4(f), *p* < 0.01). These results above indicated that the knockdown of THBS2 suppressed the osteogenic differentiation of VICs yet overexpression of THBS2 led to a promotion.

### 3.3. Akt/NF-*κ*B Pathway Participated in THBS2-Induced Osteogenic Differentiation of VICs

**Figure 5:**
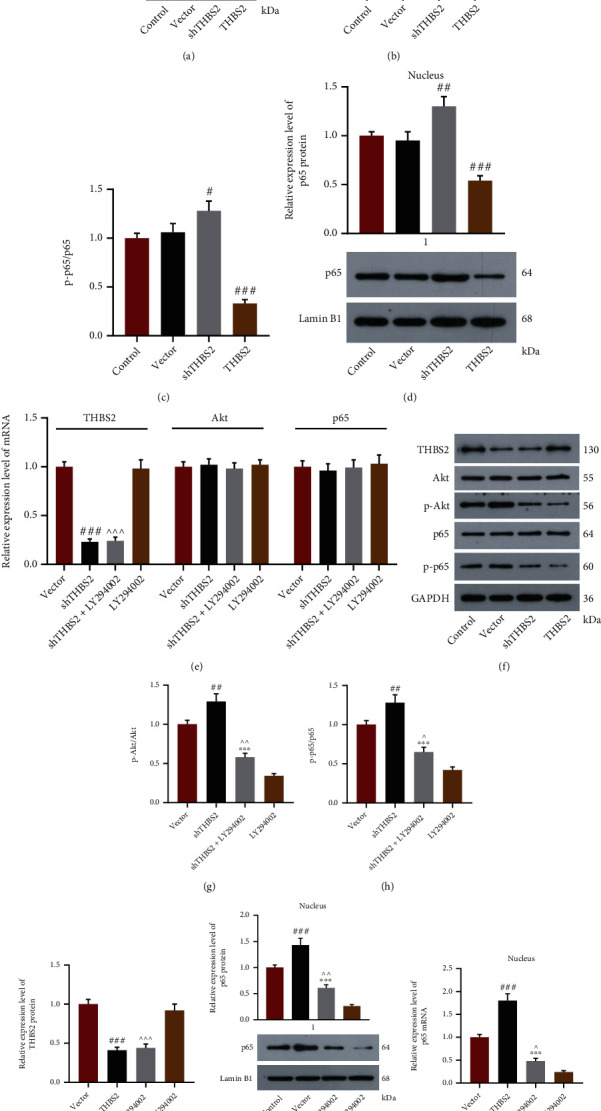
THBS2 negatively regulated Akt/NF-*κ*B signaling pathway during OD procedure of VICs. (a–c) Representative images of protein bands during Western blot (a) and the levels of p-Akt/Akt (b) and p-p65/p65 (c) of VICs transfected with shTHBS2 and THBS2 overexpression plasmid were assessed by Western blot after the induction of OD. GAPDH was a loading control. (d) Relative expression of p65 in nucleus of VICs transfected with shTHBS2 and THBS2 overexpression plasmid was assessed by Western blot after the induction of OD. Lamin B1 was a loading control. (e) Relative mRNA expressions of THBS2, Akt, and p65 in VICs transfected with shTHBS2 and treated with LY294002 were tested by qRT-PCR after the induction of OD. GAPDH was a loading control. (f) Representative images of protein bands during Western blot. (g and h) The levels of p-Akt/Akt (g) and p-p65/p65 (h) of VICs transfected with shTHBS2 and treated with LY294002 were determined through Western blot after the induction of OD. GAPDH was a loading control. (i) Relative protein expression of THBS2 in VICs transfected with shTHBS2 and treated with LY294002 was determined through Western blot after the induction of OD. GAPDH was a loading control. (j) and (k) Relative mRNA (j) and protein (k) expressions of p65 in nucleus of VICs transfected with shTHBS2 and treated with LY294002 were detected by Western blot after the induction of OD. Lamin B1 was a loading control. ^#^*p* < 0.05, ^##^*p* < 0.01, ^###^*p* < 0.001 vs. vector group; ^∗∗∗^*p* < 0.01 vs. shTHBS2 group; ^∧^*p* < 0.05; ^∧∧^*p* < 0.01; ^∧∧∧^*p* < 0.001 vs. LY294002 group. All experiments were repeated independently at least three times. Data were performed as the means ± standard deviation. Abbreviations: THBS2: thrombospondin 2; OD: osteogenic differentiation; VICs: valve interstitial cells; p-: phosphorylation-; shTHBS2: short hairpin THBS2; GAPDH: glyceraldehyde-3-phosphate dehydrogenase; qRT-PCR: quantitative reverse transcription-polymerase chain reaction.

**Figure 6:**
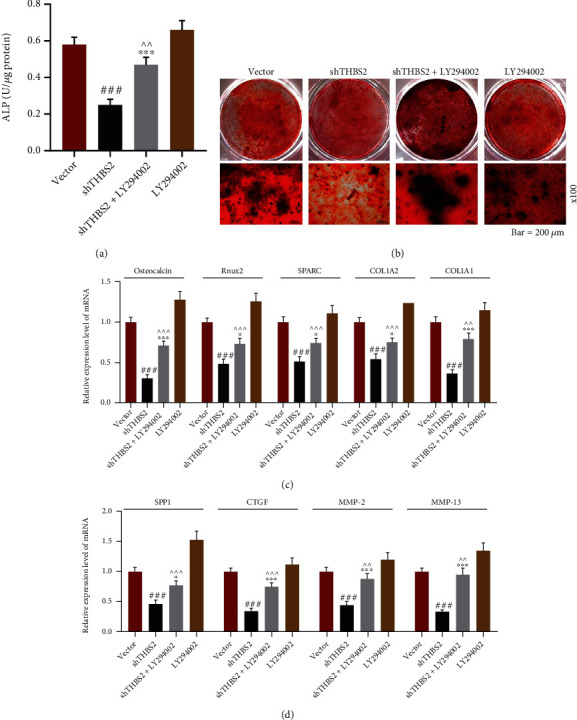
THBS2 mediated the OD of VICs via inhibition of Akt/NF-*κ*B signaling pathway. (a) ALP activity of VICs transfected with shTHBS2 and treated with LY294002 was detected through colorimetry after the induction of OD. (b) Calcic nodule formation of VICs transfected with shTHBS2 and treated with LY294002 was measured by Alizarin red staining after the induction of OD (scale: 200 *μ*m; magnification: ×100). (c) and (d) Relative mRNA expressions of osteocalcin, Runx2, SPARC, COL1A2, and COL1A1 (c) and SPP1, CTGF, MMP-2, and MMP-13 (d) in VICs transfected with shTHBS2 and treated with LY294002 were determined through qRT-PCR after the induction of OD. GAPDH was a loading control. ^###^*p* < 0.001 vs. vector group; ^∗^*p* < 0.05, ^∗∗∗^*p* < 0.01 vs. shTHBS2 group; ^∧∧^*p* < 0.01; ^∧∧∧^*p* < 0.001 vs. LY294002 group. All experiments were repeated independently at least three times. Data were performed as the means ± standard deviation. Abbreviations: THBS2: thrombospondin 2; OD: osteogenic differentiation; VICs: valve interstitial cells; ALP: alkaline phosphatase; shTHBS2: short hairpin THBS2; Runx2: Runt-related transcription factor-2; SPARC: secreted protein acidic and rich in cysteine; COL1A: collagen type I alpha; SPP1: secreted phosphoprotein 1; CTGF: connective tissue growth factor; MMP-: matrix metalloproteinase-; qRT-PCR: quantitative reverse transcription-polymerase chain reaction; GAPDH: glyceraldehyde-3-phosphate dehydrogenase.

The results of Western blot demonstrated that compared with cells transfected with empty vector, VICs transfected with shTHBS2 presented an increased expressions of p-Akt/Akt, p-p65/p65, and nuclear p65 mRNA (Figures 5(a)–5(d), *p* < 0.05), while the opposite results were shown in cells transfected with THBS2 overexpression plasmid (Figures 5(a)–5(d), *p* < 0.001). To explore whether Akt/NF-*κ*B pathway participated in THBS2-induced osteogenic differentiation, VICs were transfected with shTHBS2 and treated with Akt inhibitor LY294002. The THBS2 expression in shTHBS2 group was confirmed to be lower than that in vector group (Figures 5(e), 5(f), and 5(i), *p* < 0.001), while THBS2 expression in shTHBS2+LY294002 group was also lower than that in LY294002 group (Figures 5(e), 5(f), and 5(i) , *p* < 0.001). Moreover, our experimental data exhibited that the expressions of p-Akt/AKT, p-p65/p65, and nuclear p65 in shTHBS2+LY294002 group were lower than those in shTHBS2 group (Figures 5(f)–5(h) and 5(j) and 5(k), *p* < 0.001) yet were higher than those in LY294002 group (Figures 5(f)–5(h) and 5(j) and 5(k), *p* < 0.05). On the contrary, ALP activity, the colored area of Alizarin staining, and the mRNA levels of osteocalcin, Runx2, SPARC, COL1A2, COL1A1, SPP1, CTGF, MMP-2, and MMP-13 in shTHBS2+ LY294002 group were higher than those in shTHBS2 group (Figures 6(a)–6(d), *p* < 0.05) yet were lower than those in LY294002 group (Figures 6(a)–6(d), *p* < 0.01). What we discovered, herein, revealed that THBS2 advanced the osteogenic differentiation of VICs via inhibition of Akt/NF-*κ*B signaling pathway.

## 4. Discussion

With the aging of population, CAVD, as the most prevalent valve disease, increasingly afflicts the elderly people without effective medical treatment to prevent or reverse its initiation and advancement [3].The aberrantly elevated expression of THBS2 has been viewed to be associated with several processes related to the progression of CAVD, including inflammation, ECM remodeling, and neovascularization [7]. Consistent with the researches on gene identification in CAVD and the increasing level of THBS2 in human fibrosclerosis and AS [4, 7], the bioinformatics analysis in our study exhibited that THBS2 was higher-expressed in AS samples than in AVSc samples. As compared with normal tissues, a higher immunoreactivity of THBS2 and an elevated mRNA level of THBS2 were observed in CAVD tissues, the results of which were additionally confirmed by the following experimental results. These findings suggested that THBS2, which might serve as a potential biomarker for CAVD, indeed had a certain relationship with the occurrence and progression of CAVD.

Despite the complicated pathophysiology, the mechanism of CAVD is similar to physiological bone formation and remodeling [5]. VICs, a major cell type of AV, have been believed to promote CAVD via calcium deposition, as evidenced by their transformation into osteoblast-like ones [3, 5, 8, 27, 28]. To explore whether THBS2 participated in the calcification of VICs, the osteogenic differentiation of VICs was firstly induced as required. During the progression of osteogenic differentiation, the accumulation of THBS2 was first observed, in parallel with the rise of ALP activity and calcified nodule formation, which is considered the marker of osteogenic differentiation [3]. Besides, the expression levels of osteocalcin, Runx2, SPARC, COL1A2, COL1A1, SPP1, CTGF, MMP-2, and MMP-13 were increased as well with the time of osteogenic differentiation. Generally synthesized by osteoblasts and odontoblasts, osteocalcin is a noncollagenous bone matrix protein mediating bone mineralization and widely accepted as a good marker of mature osteoblasts [33–35]. Runx2 is a transcription factor essential for both osteogenic differentiation and osteogenesis [36, 37]. SPARC is a matricellular protein modulating the interaction between cells and ECM [38] and is an excellent marker of osteoblast maturation, which is highly specific for bone mineralization and similar to osteocalcin [33]. COL1A2 and COL1A1 belong to the fibrous collagen family and are responsible for the formation of type I collagen, the main component of osteoblasts matrix [39–41]. SPP1 is a matricellular protein and late marker for osteoblasts intimately connecting with bone matrix mineralization [42]. CTGF is reported to be a profibrotic growth factor which fulfills a vital function on pathogenesis of tissue fibrosis [43]. MMP-2 and MMP-13 are two members of MMPs which are a group of zinc containing enzymes implicated in the remodeling of ECM [44]. Both of them are verified to have a profound impact on bone formation and remodeling [45, 46]. To sum up, THBS2 might have an association with the osteogenic differentiation of VICs.

For a further investigation on the role of THBS2 in the osteogenic differentiation of VICs, VICs were transfected with shTHBS2 and THBS2 overexpression plasmid to construct the models of THBS2 silencing and overexpression prior to the induction of osteogenic differentiation. Previous reporters have shown that THBS2 could regulate MMP-2 and MMP-13 to promote tumor metastasis of prostate cancer and lung cancer [3, 24, 25]. Similarly, our work validated that the knockdown of THBS2 repressed ALP activity, calcified nodule formation, and the levels of factors related to osteogenic differentiation (osteocalcin, Runx2, SPARC, COL1A2, COL1A1, SPP1, and CTGF) and ECM (MMP-2 and MMP-13), while overexpressed THBS2 did the opposite. These results thus led us to conclude that THBS2 overexpression played a positive role in CAVD development via inducing the osteogenic differentiation of VICs.

AKT/NF-*κ*B pathway has been reported to be involved in CAVD [47], and cardamonin inhibits the phenotypical calcific transformation of human VICs by mediating the inactivation of the NF-*κ*B/NLRP3 inflammasome [48]. It has been revealed that the effects of THBS2 on upregulating MMP-13 and promoting the mobility of lung cancer cells were realized via the integrin *α*v*β*3/FAK/Akt/NF-*κ*B pathway [25]. Moreover, the inactivation of Akt/NF-*κ*B pathway was regarded to be involved in the regulation of THBS2 in CAVD [7], and the results of our bioinformatics analysis also demonstrated the participation of PI3K-Akt signaling pathway in CAVD. Thus, we presumed that THBS2 affected the osteogenic differentiation of VICs by Akt/NF-*κ*B signaling pathway. Based on the data of Western blot, we viewed that silenced THBS2 facilitated the phosphorylation of Akt and p65 as well as the elevation of nuclear p65 expression, whereas overexpressed THBS2 did conversely. Those data suggested that overexpressed THBS2 suppressed Akt/NF-*κ*B pathway through inhibiting phosphorylation of Akt and p65 and nuclear translocation of p65 during the osteogenic differentiation of VICs. To test the implication of Akt/NF-*κ*B signaling pathway in calcified VICs induced by THBS2, VICs were transfected with shTHBS2 and treated with Akt inhibitor LY294002 before the induction of osteogenic differentiation. The succeeding results underlined that LY294002 partly reversed the effect of THBS2 knockdown on promoting the phosphorylation of Akt and p65 and the nuclear translocation of p65 yet repressing ALP activity, calcified nodule formation, and the levels of factors related to osteogenic differentiation and ECM, proving that THBS2 mediated osteogenic differentiation of VICs via Akt/NF-*κ*B signaling pathway.

In a word, the present paper demonstrates that THBS2 is high-expressed in CAVD and its overexpression could promote the development of CAVD through inducing the osteogenic differentiation of VICs. Furthermore, such effects of THBS2 on VICs are achieved by the inhibition of Akt/NF-iB pathway. Collectively speaking, this work might provide a promising biomarker and therapeutic target for CAVD.

## Figures and Tables

**Table 1:** Primer sequences used for quantitative reverse transcription-polymerase chain reaction (qRT-PCR).

Target gene	Primers, 5'-3'

THBS2	Forward: ATAGACAGCTTCGCTCTGGAC
	Reverse: CAAACCCCTGAAGTGACTCTC
Osteocalcin	Forward: GGCGCTACCTGTATCAATGG
	Reverse: GTGGTCAGCCAACTCGTCA
Runx2	Forward: TCAACGATCTGAGATTTGTGGG
	Reverse: GGGGAGGATTTGTGAAGACGG
SPARC	Forward: CCCATTGGCGAGTTTGAGAAG
	Reverse: CAAGGCCCGATGTAGTCCA
COL1A2	Forward: GAGCGGTAACAAGGGTGAGC
	Reverse: CTTCCCCATTAGGGCCTCTC
COL1A1	Forward: GAGGGCCAAGACGAAGACATC
	Reverse: CAGATCACGTCATCGCACAAC
SPP1	Forward: CTCCATTGACTCGAACGACTC
	Reverse: CAGGTCTGCGAAACTTCTTAGAT
CTGF	Forward: AAAAGTGCATCCGTACTCCCA
	Reverse: CCGTCGGTACATACTCCACAG
MMP-2	Forward: GATACCCCTTTGACGGTAAGGA
	Reverse: CCTTCTCCCAAGGTCCATAGC
MMP-13	Forward: TCCTGATGTGGGTGAATACAATG
	Reverse: GCCATCGTGAAGTCTGGTAAAAT
Akt	Forward: TCCTCCTCAAGAATGATGGCA
	Reverse: GTGCGTTCGATGACAGTGGT
p65	Forward: ATGTGGAGATCATTGAGCAGC
	Reverse: CCTGGTCCTGTGTAGCCATT
GAPDH	Forward: CTGGGCTACACTGAGCACC
	Reverse: AAGTGGTCGTTGAGGGCAATG

## Data Availability

The analyzed data sets generated during the study are available from the corresponding author on reasonable request.
